# Prevalence of Segond fractures associated with anterior cruciate ligament injuries and their influence on knee joint stability; A case-control study

**DOI:** 10.1186/s12891-022-05127-w

**Published:** 2022-02-24

**Authors:** Ryotaro Kumahara, Yuka Kimura, Shizuka Sasaki, Eiji Sasaki, Shugo Maeda, Harehiko Tsukada, Yuji Yamamoto, Eiichi Tsuda, Yasuyuki Ishibashi

**Affiliations:** 1 Department of Orthopaedic Surgery, Hirosaki University Graduate School of Medicine, 5 Zaifu-cho, Hirosaki, 036-8562 Japan; 2 Department of Orthopaedic Surgery, Aomori Rosai Hospital, Hachinohe, Japan; 3 Department of Orthopaedic Surgery, Aomori City Hospital, Aomori, Japan; 4 Department of Rehabilitation Medicine, Hirosaki University Graduate School of Medicine, Hirosaki, Japan

**Keywords:** Segond fracture, Anterior cruciate ligament injury, Anterior cruciate ligament reconstruction, Knee joint stability, Knee

## Abstract

**Background:**

The purpose of this study was to determine the prevalence of Segond fractures and to compare knee stability between patients undergoing primary anterior cruciate ligament (ACL) reconstruction with and without Segond fractures pre- and postoperatively.

**Methods:**

A total of 712 patients who underwent primary ACL reconstruction between 2014 and 2019. Exclusion criteria included patients with multi-ligament knee injuries, skeletally immature patients, osteoarthritis in the knee, combined surgery of high tibial osteotomy, lack of data, and loss to follow-up for at least 2 years. Segond fractures were confirmed using plain radiography, computed tomography (CT), and magnetic resonance imaging (MRI). Patients with Segond fractures were classified into Group S and without Segond fractures into Group N. Pre- and postoperative Lachman grades, pivot-shift grades, and assessment of side-to-side differences in anterior stability were evaluated.

**Results:**

Five hundred and forty patients included in this study. There were 22 patients with Segond fractures. Of these, all 22 cases (4.1%) were identified on CT, but only 20 cases (3.7%) were identified on MRI and 18 cases (3.3%) on plain radiographs. There was no significant difference in preoperative Lachman grade or pivot-shift grade between Groups S and N (*p* = 0.662, *p* = 0.677, respectively). There was no significant difference in postoperative Lachman grade or pivot-shift grade between Groups S and N (*p* = 0.685, *p* = 0.390, respectively). There were no significant differences in preoperative (*p* = 0.398) or postoperative (*p* = 0.546) side-to-side differences of anterior stability between Groups S and N.

**Conclusions:**

Segond fractures were confirmed in 4.1% of the cases on CT scans among patients undergoing primary ACL reconstruction. Segond fractures did not affect preoperative or 2-year follow-up evaluations of knee stability. From these results, we concluded that Segond fractures did not affect the clinical outcomes of the primary ACL reconstruction and that it may not be necessary to treat Segond fractures.

## Background

In 1879, Paul Segond first identified a remarkably consistent avulsion fracture of the proximal tibia resulting from forced internal rotation in a cadaveric study [1]. Since then, Segond fractures have been defined as avulsion fractures of the iliotibial band (ITB) and the lateral joint capsule [2] and are recognised as typical radiographic markers of anterior cruciate ligament (ACL) injuries, indicating anterolateral rotatory instability of the knee joint [3–5]. Recent evidence has suggested that Segond fractures are bony avulsions of the anterolateral complex (ALC), which includes the distal ITB including proximal and distal capsule-osseous fibres (proximal and distal Kaplan’s fibres), portions of the lateral meniscocapsular junction, and the anterolateral ligament (ALL) [6].

The anatomical function of ALC and the effect of ALC ruptures have received significant attention. Based on an anatomical study, the hypothesised function of the ALL is to control internal tibial rotation and thus affect the pivot-shift phenomenon [3]. In biomechanical studies, the ALL acts as a restraint for internal rotation of the tibia and affects the pivot shift in ACL-deficient knees [3, 7, 8].

However, there is still no consensus regarding ALL reconstruction. The purpose of this study was to determine the prevalence of Segond fractures and the stability of pre- and postoperative knees without ALL reconstruction and repair of Segond fractures. We hypothesised that the presence of Segond fractures with ACL injuries would affect pre- and postoperative knee stability.

## Methods

The study design was approved by the ethics committee of the institutions, and all patients signed an informed consent document. We retrospectively evaluated 712 patients who underwent primary ACL reconstruction in our hospital and two affiliated hospitals between 2014 and 2019. Exclusion criteria included: 1) patients with multi-ligament knee injuries, except for those with injury to the medial collateral ligament (MCL) in addition to the ALL; 2) skeletally immature patients with an open physis of the femur and tibia detected using magnetic resonance imaging (MRI) [9]; 3) osteoarthritis in the knee (Kellgren-Lawrence grade > 3); 4) combined surgery of high tibial osteotomy; 5) lack of data or imaging; and 6) patients lost to follow-up for at least 2 years.

### Radiological evaluation

A Segond fracture is defined as any bony avulsion of the proximal tibia confirmed on anteroposterior plain radiographs, computed tomography (CT) scans, or MRI. Plain radiographs and MRI were used preoperatively for the diagnosis of ACL injuries. MRI scans evaluated only the osseous fragment and no other structures such as ALL injuries and anterolateral structures. In our institute, CT were routinely performed the day after surgery to assess the tunnel positions. Fracture sizes were determined using CT by measuring the maximum proximal-distal length, medial-lateral (ML) width, and anterior-posterior (AP) width of the avulsed fragment. Additionally, using coronal-plane CT scans, the maximal displacement was measured in millimetres from the most proximal edge of the lateral articular surface and the presumed centre of the fracture bed on the proximal tibia. Spontaneous healing of the Segond fracture was confirmed on MRI at 3 months postoperatively, which is routinely performed in our institute to assess the graft and meniscus.

### Surgical technique and postoperative rehabilitation

The senior author performed or directly supervised (guided all important surgical decisions) ACL reconstruction in all patients. An arthroscopic examination was performed first, followed by an assessment of the ACL and meniscus by probing. ACL reconstructions were performed with a rectangular single-bundle technique using bone tendon-bone grafts [10] and a double-bundle technique using hamstring tendon grafts [11, 12]. Small and stable meniscal tears were left in situ, unstable tears were repaired, and severe degeneration or flap tears were partially resected. Segond fractures were not repaired in any patient, and ALLs were not reconstructed. Postoperative rehabilitation followed the same protocol for all the patients. After ACL reconstruction, patients began crutch-assisted partial weightbearing, range-of-motion exercises, and isometric muscle-strengthening exercises the day after surgery. Full weightbearing and closed kinetic chain exercises were allowed starting 7 days postoperatively. Open kinetic-chain exercises and running were performed 3 months postoperatively. Sport-specific training was allowed 5–6 months postoperatively, and patients were allowed to return to sports activities 6–9 months postoperatively.

### Clinical evaluations

In our institution, physical assessments for the stability of the knee joint are routinely performed before surgery and 2 years after surgery. The mechanisms of injury (contact or non-contact) were recorded from medical records. A contact injury was defined as an injury in which an external force directly impacted the level of the knee, whereas non-contact injuries included all other mechanisms, including landing or pivoting injuries. The presence of knee recurvatum was evaluated preoperatively and was noted when it was greater than 10° on the contralateral side. Knee laxity was usually evaluated using Lachman and pivot-shift test grades and by side-to-side differences in the anterior tibial translation measured preoperatively and at the 2-year follow-up evaluation with a KT-1000 arthrometer (formerly MEDmetric Corp., San Diego, CA). The number of ipsilateral ACL graft ruptures during the follow-up period was recorded for both groups. Clinical evaluations were performed by five experienced orthopaedic surgeons who were blinded to the presence of Segond fractures.

### Statistical methods

All statistical analyses were performed using SPSS software (version 12.0 J; IBM Corp., Armonk, NY). We divided all subjects into two groups: patients with Segond fractures were classified into Group S, and patients without Segond fractures were classified into Group N. The normality of the variables was determined using the Shapiro-Wilk test. Demographic data (age, body mass index [BMI]) and side-to-side differences in anterior tibial translation were compared between groups using the Mann-Whitney U test. Demographic data (sex, knee recurvatum, mechanisms of injury, graft type), postoperative Lachman test grades, and graft ruptures were compared between the groups using Fisher’s exact test. The prevalence of meniscus injuries, preoperative Lachman test grades, and pre- and postoperative pivot-shift test grades were compared between the groups using the χ^2^ test. Differences were considered statistically significant at *p* < 0.05. Post-hoc power analysis showed a power of 95.6% with an α-value of 0.05, demonstrating a large effect size (r = 0.8).

## Results

**Fig. 1:**
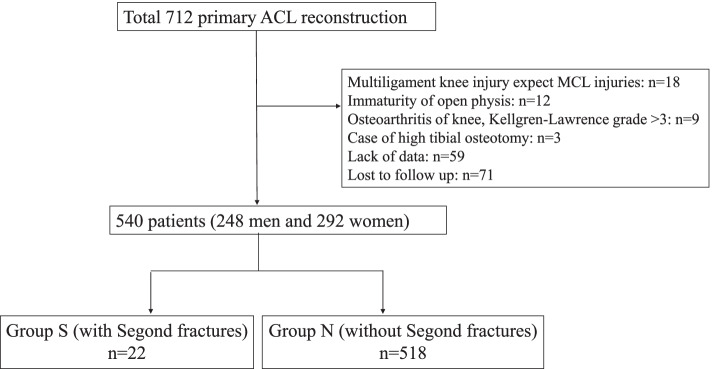
Patient flowchart

A total of 540 patients (248 men and 292 women, average age: 24.8 ± 11.4 years) were included in this study (Fig. 1). There were 22 patients with Segond fractures (Group S) (Table 1). Of these, all 22 cases (4.1%) were identified on CT, but only 20 cases (3.7%) were identified on MRI and 18 cases (3.3%) on plain radiographs.

**Table 1:**
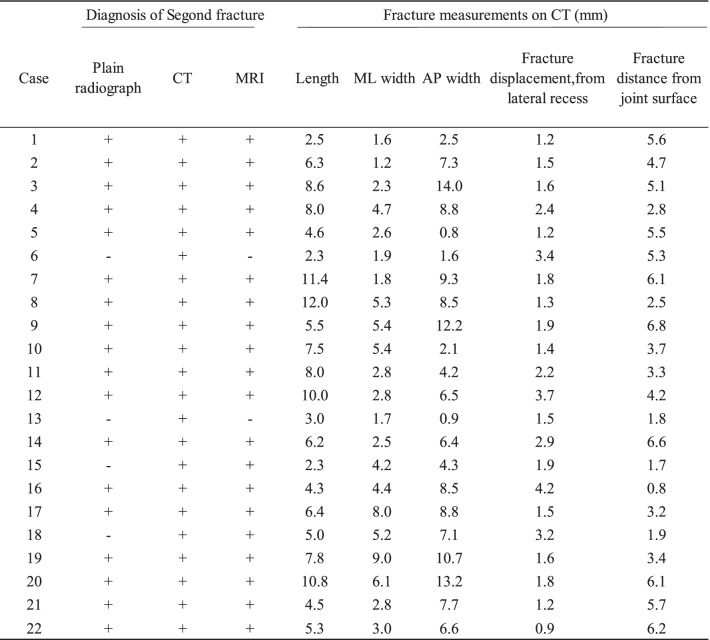
Diagnosis of Segond fracture by modality and measurement data using CT

*ML* Medial-lateral, *AP* Anterior-posterior, *CT* Computed tomography, *MRI* Magnetic resonance image

Demographic data of the patients in Groups S and N are shown in Table 2. There were significant differences in BMI (*p* = 0.002); however, there were no significant differences in age (*p* = 0.956), sex (*p* = 0.069), mechanism of injury (*p* = 0.248), knee recurvatum (*p* = 0.509), prevalence of meniscus injuries (*p* = 0.810) or graft type (*p* = 0.131) between Group S and Group N (Table 2). Postoperative MRI was available for all patients, and the spontaneous healing rate was 100%. Pre- and postoperative knee joint stability values are shown in Table 3. There were no significant differences between Group S and Group N in the preoperative evaluations and at the 2-year follow-up in terms of knee stability. In Group N, 39 (7.5%) graft ruptures were observed, compared with 0 graft ruptures in Group S (Table 3), but there were no significant differences between the two groups.

**Table 2:**
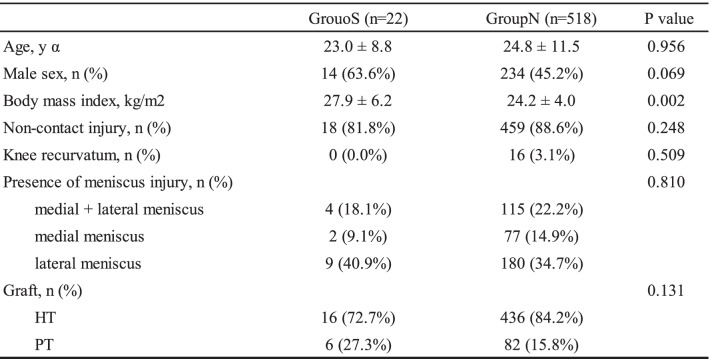
Demographic data of the study patients

α Data are presented as mean ± SD or n (%). *HT* Hamstring tendon, *PT* Patellar tendon

**Table 3:**
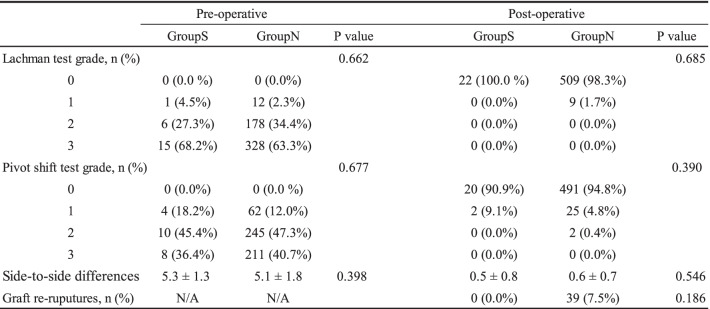
Pre- and postoperative knee joint stability

α Data are presented as mean ± SD or n (%)

## Discussion

The most important finding in this study was that the prevalence of Segond fractures in primary ACL reconstruction was 4.1% and Segond fractures did not affect pre- or postoperative knee stability. There was a significant difference in BMI; however, there were no significant differences in the mechanisms of injury and prevalence of meniscal tears between those with and without Segond fractures. This was not consistent with our hypothesis that the presence of a Segond fracture with concomitant ACL injury would increase knee laxity preoperatively and after ACL reconstruction. In this study, the spontaneous healing rate was 100% on postoperative MRI, which suggests that Segond fractures allow for osseous healing of the ALC.

The prevalence of Segond fractures in patients sustaining an ACL injury varies [2, 4, 5, 13–17]. This variation in the prevalence of Segond fractures might be caused by the use of different diagnostic tools in a diverse patient population. Segond fractures have been diagnosed using plain radiographs, CT, MRI, and ultrasonography [18]. The prevalence of Segond fractures on plain radiographs was 6–9% [13, 16, 17, 19], and confirmation by MRI ranged from 2.6–8.9% [2, 14, 17]. Yoon et al. [17] reported that Segond fractures were confirmed in 6.7% of cases using plain radiographs, 4.2% using MRI, and 8.9% using CT, and that the prevalence determined with CT was higher than that determined with plain radiographs. Segond fractures are less detectable on MRI because the small size of the fragments can be concealed by swelling and hematomas of the knee in acute injuries [18]. Gaunder et al. [13] reported that Segond fracture fragments typically measure 6.6 mm in length and 2.3 mm in width on plain radiographs and are seen 9.7 mm below the joint line. From the results of this study on CT measurements, the size of fragments and displacement were similar to those of a previous study. The strength of the current study was the use of CT and MRI in addition to plain radiographs for the diagnosis of Segond fractures, although ultrasonography was not evaluated. In addition, MRI was used to evaluate fracture healing, and the routine CT and MRI images in this investigation were of high quality.

In our cohort, the incidence of Segond fractures identified by CT scans (4.1%) in the Japanese population was relatively lower than that in previous reports [2, 4, 5, 13–16]. One of the reasons for this may be the difference in the prevalence of ALL and morphological variations in the Japanese population. Watanabe et al. [20] examined the prevalence of ALL in 94 cadaveric knees and reported that ALL was seen in 20.2% of Japanese people, which was significantly lower than previous reports in Western populations. Although there was a large age difference between the cadaveric study and the present study, this morphological variation may explain why the percentage of Segond fractures in the Japanese population was found to be lower than that in Western populations [20].

It was previously reported that the presence of a Segond fracture is more common in male patients [6, 13]. In this study, there were no significant difference by sex, but there were slightly more males (63.6%). Although contact ACL injuries are considered to be caused by higher-energy damage than non-contact injuries, there was no significant difference in the prevalence of Segond fractures between contact and non-contact injuries in this study. More data are needed to detect sex-specific differences in the prevalence of Segond fractures, which may provide insight into sex-specific mechanisms of injury.

Recent evidence suggests that ALL functions as a secondary stabiliser to the ACL in resisting anterior tibial translations and internal tibial rotations to prevent the pivot-shift phenomenon [3, 7, 8, 21]. As assessed on clinical examination, this study found no difference in knee stability between the two groups at their preoperative evaluations or at the final follow-up. Yoon et al. [17] and Melugin et al. [22] investigated the effect of Segond fractures on the outcome of primary ACL reconstruction, and both concluded that the presence of a Segond fracture did not affect knee laxity in patients with ACL tears and that there were no significant differences in knee stability and clinical scores between patients with or without a Segond fracture after ACL reconstruction. Gaunder et al. [13] compared the incidence of Segond fractures in patients undergoing primary ACL reconstruction with those undergoing revision reconstruction and concluded that Segond fracture was not a risk factor for failure after ACL reconstruction. Slagstad et al. [19] investigated 101 patients with Segond fractures, the largest Segond fracture cohort, and reported that the spontaneous healing rate was 36%; however, the presence of Segond fracture was not related to any increase in the risk of revision surgery.

Some authors have proposed that the presence of a Segond fracture represents an ALL injury; therefore, it may be an indication for repair or reconstruction [23, 24]. Sonnery-Cottet et al. [24] suggested that a combined ACL and ALL reconstruction should be considered to improve the control of rotational stability in patients at high risk of graft failure associated with Segond fractures, chronic ACL injuries, and high levels of activity. In contrast, several studies have found that a combined ACL and ALL reconstruction is not necessary, because the Segond fracture does not confer a high risk of graft failure compared to treatment with ACL reconstruction alone [13, 22, 25]. Moreover, postoperative radiographs revealed that Segond fractures healed in 90% of patients and that the patients undergoing ACL reconstruction with or without a Segond fracture had similar pivot-shift test results and graft-failure rates [10]. In addition, Shaikh et al. [6] reported successful treatment of Segond fractures that continued for decades by the natural healing response in the absence of a repair. In this study, the spontaneous healing rate was 100% on postoperative MRI. Our study evaluated patients with Segond fractures and should be interpreted as separate from the results of patients with ACL injuries and concomitant ALL injuries without Segond fractures. However, patients with a combined ACL injury and Segond fractures can have outcomes comparable to those of patients with an ACL injury and no Segond fractures when treated with ACL reconstruction alone. These results suggest that ALL repair or reconstruction for Segond fractures may not be necessary.

## Limitations

This study had several limitations. First, this was a retrospective study conducted in several facilities. However, the number of subjects was sufficient in the post-hoc analysis. One of the problems with multicentre studies is that, although the surgical method was consistent, differences of clinical outcome among surgeons might appear. Second, we only evaluated the Segond fractures and did not evaluate ALL injuries or anterolateral structures on MRI. Ferretti et al. [26] investigated that the ALL abnormalities on MRI of both knees in 34 acute ACL-injured patients. And they reported that 88.2% of patients had ALL abnormality which was associated with lateral joint capsular tears in ACL injured knee. Therefore, Group N in this study may have included patients with ALL injuries without Segond fractures. Third, the current study lacked data regarding return to sports activities and patient-reported functional outcome measures. Finally, a quantitative measurement device was not used to grade the Lachman and pivot-shift test results. This limitation can be resolved using a navigation system [27, 28] or other electromagnetic systems [29].

## Conclusion

Segond fractures were confirmed in 4.1% of the cases on CT scans among patients undergoing primary ACL reconstruction. Segond fractures did not affect preoperative or 2-year follow-up evaluations of knee stability. From these results, we concluded that Segond fractures did not affect the clinical outcomes of the primary ACL reconstruction and that it may not be necessary to treat Segond fractures.

## Data Availability

All data generated or analysed in this study are included in this published article.

## Abbreviations

ITB: Iliotibial band
ACL: Anterior Cruciate Ligament
ALC: Anterolateral Complex
ALL: Anterolateral Ligament
MCL: Medial Collateral Ligament
MRI: Magnetic Resonance Imaging
CT: Computed Tomography
ML: Medial-lateral
AP: Anterior-posterior
BMI: Body Mass Index
